# Molecular and immunological features of TREM1 and its emergence as a prognostic indicator in glioma

**DOI:** 10.3389/fimmu.2024.1324010

**Published:** 2024-02-02

**Authors:** Lin Zhang, Xun Qu, Yangyang Xu

**Affiliations:** ^1^Department of Clinical Laboratory, Qilu Hospital of Shandong University, Jinan, China; ^2^Institute of Basic Medical Sciences, Qilu Hospital of Shandong University, Jinan, China; ^3^Department of Neurosurgery, Qilu Hospital of Shandong University and Institute of Brain and Brain-Inspired Science, Shandong University, Jinan, China

**Keywords:** glioma, TREM1, prognosis, macrophage, tumor microenvironment

## Abstract

Triggering receptor expressed on myeloid cells 1 (TREM1), which belongs to the Ig-like superfamily expressed on myeloid cells, is reportedly involved in various diseases but has rarely been studied in glioma. In this study, the prognostic value and functional roles of TREM2 in glioma were analyzed. TERM1 was observed to be significantly upregulated in GBM compared to in other grade gliomas and was associated with poor prognosis. Increased TREM1 accompanied distinct mutation and amplification of driver oncogenes. Moreover, gene ontology and KEGG analyses showed that TREM1 might play a role in immunologic biological processes in glioma. TREM1 was also found to be tightly correlated with immune checkpoint molecules. xCell research revealed a link between TREM1 expression and multiple immune cell types, especially monocytes and macrophages. Single-cell analysis and immunofluorescence results showed that macrophages expressed TREM1. *In vitro*, inhibition of TREM1 signaling could result in a decrease in tumor-promoting effects of monocytes/TAMs. In summary, TREM1 may be a potential independent prognostic factor and immune target, which might provide new avenues to improve the efficacy of immunotherapy in glioma patients.

## 1 Introduction

Glioma is the most prevalent and lethal type of primary malignant tumor in the central nervous system (CNS) (1). Although it has made great advances in multimodal therapy, including total resection plus adjuvant radio-chemotherapy, electric field therapy and targeted drug therapy, the five-year survival rate is still less than 10% (2). Glioma patients succumb in large part due to the rapid growth of tumors, high invasiveness, and resistance to treatments (3). It has been demonstrated that tumor microenvironment (TME), which contains tumor cells, stromal cells, infiltrating immune cells, soluble factors, blood vessels and extracellular matrix, plays a critical role in supporting the progression of glioma (4, 5).

With the deep-going research, clinical trials of immunotherapies that target the immune components of TME have shown exceptional promise for the treatment of cancer (6). A large number of studies have confirmed that a breakthrough in cancer immunotherapy is emerging with drugs that target immune checkpoint molecules. Under physiological circumstances, immune checkpoints play a crucial role in maintaining self-tolerance and in tuning the duration and amplitude of immune responses. Through the breakdown of this delicate balance, cancer is able to counteract immune system attacks by usurping the mechanisms that usually prevent autoimmunity. Immune checkpoint inhibitors (ICIs) and monoclonal antibodies (mAbs) targeting inhibitory receptors have been subjected to clinical trials, and striking success has been achieved across multiple solid tumors (7–9). However, monotherapy with immune checkpoint blockade for most gliomas does not seem to induce durable antitumor responses (10). It has been indicated that ICI-combined drug therapy can exhibit remarkable advantages and efficacy. In pancreatic ductal adenocarcinoma, IL-17 blockade increased immune checkpoint blockade (PD-1, CTLA4) sensitivity (11). In breast and colon cancer-bearing mouse models, CCR4 inhibition enhanced the antitumor effect of anti-CTLA-4 and resulted in long-lasting immunity (12). These exciting results inspired us to explore other potential immunological targets in the glioma microenvironment.

The TREM receptor family, which belongs to the Ig-like superfamily, has been demonstrated to be involved in inflammatory diseases, autoimmune disorders, and cancers (13). Among them, triggering receptor expressed on myeloid cells-1 (TREM1) is the first identified and best-characterized family member. TREM1 plays roles in the release of proinflammatory cytokines, T-cell differentiation, and effector responses (14). Studies have shown that TREM1 is associated with the occurrence of immune-related inflammatory diseases, such as arthritides, pancreatitis, and bowel inflammatory disorders (15, 16). TREM1 mRNA and protein upregulation was accompanied by an increased inflammatory response and disease severity in mouse models of colitis, which were attenuated in TREM1-deficient mice (17). Recent investigations have highlighted the important influence of TREM1 signaling on inflammation-mediated carcinogenesis. High TREM1 expression levels in lung adenocarcinoma and hepatocellular carcinoma were suggested to be independent predictors of tumor progression and poor prognosis (18, 19). It could be possible to diagnose early cancers in a broad population by assessing sTREM concentrations levels in biological fluids (20). A TREM1 inhibitor could sensitize tumors to immunotherapy in a model of hepatocellular carcinoma by synergistically reversing immunosuppression and reactivating effector T cells to exert tumor cell cytotoxicity (21). Therefore, TREM1 may be a novel and attractive immune target and may exhibit new avenues to overcome the low response rate of glioma patients to current immunotherapies.

To date, there is no comprehensive report illustrating the immunosuppressive status with different TREM1 expression levels in glioma. In this sense, an in-depth exploration of the immunobiological processes of TREM1 based on the current genomic databases can lead to a better understanding of the complexity of the tumor microenvironment. Here, we investigated the clinical, molecular, and immunological characteristics of TREM1 in glioma. Our results demonstrated that TREM1 was highly expressed in GBM and was a valuable prognostic biomarker for glioma patients. TREM1 participated in the immunosuppressive response, correlated with tumor-associated immune cells and synergistic with several immunosuppressive members. *In vitro*, inhibition of TREM1 signaling could result in a decrease in tumor-promoting effects of monocytes/TAMs, suggesting that TREM1 might involve in the formation of immunosuppressive microenvironment. In our study, TREM1 was identified as a potential immune molecular target to further optimize immunotherapy for glioma.

## 2 Materials and methods

### 2.1 Data retrieval and preprocessing

The data included clinicopathological characteristics and transcriptome data of patients with glioma (WHO grades II-IV) obtained from the Cancer Genome Atlas (TCGA, http://cancergenome.nih.gov/) database (RNA-seq, *n* = 667), TCGA GBM database (microarray, *n=* 489), the Chinese Glioma Genome Atlas (CGGA, http://www.cgga.org.cn) database (RNA-seq, *n* = 693; microarray, *n* = 301), Gene Expression Omnibus (GEO, https://www.ncbi.nlm.nih.gov/geo/) database (GSE16011, *n* = 284), the Gill database (*n* = 92), IVY Glioblastoma Atlas Project data (http://glioblastoma.alleninstitute.org/, *n* = 270), and the Rembrandt database (https://caintegrator.nci.nih.gov/rembrandt/, *n* = 580). The scRNA-seq data of 4 GBM samples were downloaded from the Gene Expression Omnibus database (GEO, GSE84465) (22). Somatic copy number alternations (CNAs) and somatic mutations were downloaded from the TCGA data portal. GISTIC 2.0 was employed to analyze relationship between CNAs and TREM1 expression. The TREM1 protein expression level was obtained online from the Human Protein Atlas (www.proteinatlas.org).

### 2.2 Bioinformatic analysis

All transcriptome data were log2 transformed. Genes significantly related to TREM1 expression were analyzed by Pearson correlation analysis. Gene ontology (GO) analysis was performed when related gene sets were submitted to the Metascape website (https://metascape.org/gp/index.html). KEGG was performed when related gene sets were submitted to the DAVID website (http://david.abcc.ncifcrf.gov/home.jsp). Gene set enrichment analysis (GSEA) (https://www.broadinstitute.org/gsea/index.jsp) was used to identify differential phenotypes between the low and high TREM1 groups. Stromal and immune scores and tumor purity of glioma tissues were calculated using the ESTIMATE R package. T cell specific genesets were downloaded from the AmiGO2 Web portal (http://amigo.geneontology.org). Gene set variation analysis (GSVA) was used to analyze profile patterns of the transcriptome, immune function and inflammation in high or low TREM1 expression cases.

Processing and analysis of the GBM sample scRNA-seq was accordance with the previously published methods (23). In brief, the Seurat R package was employed for scRNA-seq data analysis (24). Quality control (QC) was achieved by excluding low-quality genes present in <3 cells, or low-quality cells containing <100 total identified genes, or cells containing >10% mitochondrial genes. Data normalization was performed using the normalized data function. The top 2000 highly variable genes were extracted for principal component analysis (PCA) and the top 20 principal components were used for cluster analysis. the clusters were annotated and identified based on the CellMarker database 2.0 based on DEGs.

### 2.3 Cell culture and treatments

Human glioma cell lines U118 and LN229 were purchased from ATCC, and cultured in Dulbecco’s modified Eagle medium (DMEM) supplemented with 10% fetal bovine serum (FBS) at 37°C in a humidified incubator with 5% CO_2_. For production of the conditioned medium from glioma cells (U118-CM/LN229-CM), glioma cells were cultured in 2 ml fresh serum-free 1640 medium for 24 h. Medium was collected and used as CM for further experiments.

Human peripheral blood monocytes (PBMs) from healthy donors were isolated by density-gradient centrifugation using Histopaque (Sigma-Aldrich, 10771). Monocytes were isolated using anti-CD14 microbeads (Stemcell, 17858), and more than 97% of the cells were CD14^+^ monocytes as determined by FACS analysis (data not shown). For tumor-associated macrophage (TAM) activation, CD14^+^ monocytes at 1×10^6^ cells/ml were treated for 5 days with 100 ng/ml recombinant human macrophage colony-stimulating factor (M-CSF, Peprotech, 300-25), followed by 30% U118-CM/LN229-CM for 2 days in combination with vehicle or TREM1 inhibitor. TREM1 inhibitory peptide LP17 (LQVTDSGLYRCVIYHPP, LP-17) and LP17 scramble protein (TDSRCVIGLYHPPLQVY, TY-17) were chemically synthesized by GL Biochem (Shanghai, China). LP-17 and TY-17 were used at a concentration of 200 ng/ml. TAMs were then washed with PBS and further cultured in fresh DMEM medium for 24 h. Then conditioned medium from monocyte-derived TAM (TAM-CM) was collected for further experiments.

### 2.4 Quantitative RT-PCR

Total RNA from monocytes or TAMs was extracted using the RNA-Quick Purification Kit (ES Science, China). Reverse-transcription was performed with the ReverTra Ace qPCR RT Kit (Toyobo, Japan). Quantitative RT-PCR (qRT-PCR) was conducted on the Roche Light Cycler 480 (Roche). GAPDH was used as an internal control. The primer sequences were as follows: TREM1, forward: 5’-CTT GGC AGA TAA TAA GGG ACG G-3’, reverse: 5’-CGG ACG CGC AGT AAA CCA T-3’; IL-6, forward: 5’-AAA GAG GCA CTG GCA GAA AA-3’, reverse: 5’-AGC TCT GGC TTG TTC CTC AC-3’; IL-8, forward: 5’-TTT TGC CAA GGA GTG CTA AAG A-3’, reverse: 5’-AAC CCT CTG CAC CCA GTT TTC-3’; CCL2, forward: 5’-CAG CCA GAT GCA ATC AAT GCC-3’, reverse: 5’-TGG AAT CCT GAA CCC ACT TCT-3’; CCL8, forward: 5’-TGG AGA GCT ACA CAA GAA TCA CC-3’, reverse: 5’-TGG TCC AGA TGC TTC ATG GAA-3’; GAPDH, forward: 5’-CTC TCT GCT CCT CCT GTT CGA C-3’, reverse: 5’-TGA GCG ATG TGG CTC GGC T-3’.

### 2.5 Flow cytometry

To analyze expression of TREM1 in monocytes and TAMs, cells were pre-incubated with FcR blocking reagent (Biolegend, USA, 422302) 15 min at 4°C. Then cells were stained with anti-human CD354 (TRME-1)-PE (Biolegend, USA, 314906) antibodies for 30 min at 4°C in the dark. Cells were washed with PBS twice, and suspended in PBS for detection. The samples were acquired on a FACSAria flow cytometer (BD Biosciences) and the data were analyzed with FlowJo software.

### 2.6 Migration assay

For chemotaxis assay, after 48 h of U118-CM/LN229-CM pretreatment, human circulating CD14^+^ monocytes (2×10^5^) were added into the upper compartment of the inserts (24-well plate, 5 μm pores, Costar) in 100 μl of serum-free RPMI1640. U118/LN229 conditioned medium (600 μl) was added to the lower chamber of the Transwell plate. After incubation at 37°C for 4 h, the migrated cells on the lower surface of the filter were fixed in 4% paraformaldehyde (PFA) and stained with crystal violet. Random fields of each well were photographed using a Leica microscope (Leica DFC450; Leica Microsystems).

For transwell assays, after 48 h of TAM-CM pretreatment, glioma cells (3×10^4^) were seeded into the upper chambers (8 μm pores, Costar) of 24-well plates without FBS, and TAM-CM was placed in the lower chambers. Chambers were incubated at 37°C for 24-48 h, and the cells migrating through the membranes were fixed with 4% PFA and stained with crystal violet. Images were obtained (Leica DFC450; Leica Microsystems) and cells were counted. Each experiment was performed in triplicate.

### 2.7 Enzymes linked immunosorbent assay

IL-6, IL-8, CCL2, and CCL8 levels in culture medium were measured using the human ELISA Kit (Elabscience, China) in accordance with the manufacture’s instruction.

### 2.8 Immunofluorescence

Sections (5 μm) were cut from paraffin embedded tissues and incubated with primary antibodies against TREM1 (Abcam, ab225861, 1:500) and CD163 (Origene technologies, TA506380, 1:100) at 4°C overnight and then incubated with Alexa Fluor 488-conjugated or Alexa Fluor 594-conjugated secondary antibodies (Abcam, 1:1000) for 2 h. DAPI (Beyotime, 1:1000) was used to stain the nuclei. The immunofluorescent signals were detected by fluorescence microscopy (Leica DMi8; Leica Microsystems).

### 2.9 Statistical analysis

Differences in variables between the two groups were assessed using the Student’s t-test, or a one-way analysis of variance (One-way ANOVA) with at least three groups. Kaplan-Meier survival curves were used to chart survival distributions and statistical significance between two groups was assessed by log-rank tests. Univariate and multivariate Cox regression analyses were performed to identify independent prognostic factors. R package files were used to produce the figures, including ggplot2, corrgram, circlize, nomogram, and Seurat. SPSS 19 and GraphPad Prism 8 software were employed for statistical analysis. P < 0.05 was considered statistically significant.

## 3 Results

### 3.1 Association of TREM1 expression with molecular characteristics in glioma

**Figure 1:**
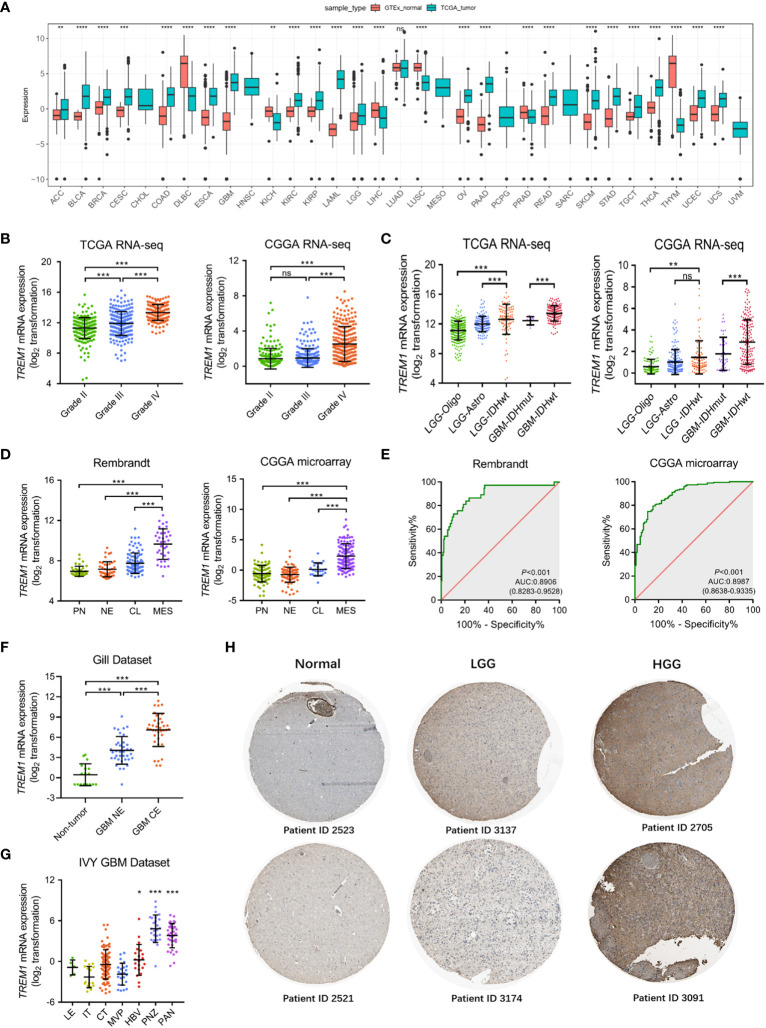
Landscape of molecular features associated with TREM1 expression. **(A)** Pan-cancer analysis of TREM1 mRNA expression in the TCGA pan-cancer datasets. **(B)** Analysis of TREM1 mRNA levels in WHO grade II-IV glioma from the TCGA and CGGA datasets (TCGA, *n* = 667; CGGA, *n* = 693). **(C)** TREM1 mRNA levels in glioma from the TCGA and CGGA datasets based on the 2016 WHO classification (TCGA, *n* = 667; CGGA, *n* = 693). **(D)** Analysis of TREM1 mRNA levels in the TCGA molecular subtypes from the Rembrandt and CGGA datasets (Rembrandt, *n* = 580; CGGA microarray, *n* = 301). **(E)** ROC curve indicating the predictive value of TREM1 expression as a biomarker of mesenchymal-subtype glioma from the CGGA and Rembrandt datasets. **(F)** Analysis of TREM1 mRNA levels in different radiographical regions of GBM in the Gill dataset (*n* = 92). **(G)** Intratumor analysis of TREM1 expression using the IVY GBM database (*n* = 270). **(H)** Analysis of TREM1 protein levels in glioma tissues of different grades from the Human Protein Atlas. Data are shown as the mean ± SD for each group. ns, no significant difference, *P <0.05, **P <0.01, ***P <0.001, ****P <0.0001. LGG-Oligo, lower-grade glioma oligodendroglioma with IDH mutation and TERT promoter mutation or 1p/19q codeletion; LGG-Astro, LGG astrocytoma with IDH mutation without TERT promoter mutation or 1p/19q codeletion, and with ATRX mutation; LGG IDHwt, LGG with wild-type IDH status; GBM IDHmut, GBM with mutant IDH status; GBM IDHwt, GBM with wild-type IDH status; PN, proneural; NE, neural; CL, classical; MES, mesenchymal; LE, Leading Edge; IT, Infiltrating Tumor; CT, Cellular Tumor; PAN, Pseudopalisading Cells Around Necrosis; PNZ, Peri-necrotic Zone; MVP, Microvascular Proliferation; HBV, Hyperplastic Blood Vessels.

It was revealed from a comprehensive analysis of TREM1 expression data obtained from The Cancer Genome Atlas Program (TCGA) and GTEx that TREM1 was highly expressed in the majority of cancers, including glioblastoma multiforme (GBM) and lower grade gliomas (LGG) (**Figure 1A**). Expression data from publicly available databases (TCGA RNA-seq, *n* = 667; CGGA RNA-seq, *n* = 693; CGGA microarray, *n* = 301; and GSE16011, *n* = 284) were further used to evaluate the expression levels of TREM1 mRNA in glioma of different WHO grades. TERM1 was observed to be increased significantly in GBM compared to grade II and grade III glioma samples (**Figure 1B** and **Supplementary Figure 1A**). Moreover, TREM1 expression was elevated with histopathologic grades (**Supplementary Figure 1B**). Gliomas are classified into five types in the 2016 WHO CNS tumor classification (1), LGG-Oligo had the lowest TREM1 expression, while GBM IDHwt showed the highest expression (**Figure 1C**). The TREM1 expression level in glioma cases with a wild-type IDH status was detected higher than those in cases with mutant IDH in both TCGA and CGGA datasets (**Supplementary Figure 1C**).

Integrated genomic- and genetic-based classification offers a new perspective for predicting outcomes in patients with different types of glioma (25). We then investigated TREM1 expression level in TCGA four molecular subtypes. As shown in **Figure 1D**, compared to the other three subtypes, TREM1 was upregulated dramatically in the mesenchymal subtype in the Rembrandt dataset, as well as in the CGGA dataset. The discrimination ability of TREM1 expression for mesenchymal subtype in all grade glioma was further assessed by Receiver operating characteristic curve (ROC) analysis. The area under the curve (AUC) of TREM1 expression was 0.8906 in the Rembrandt cohort (**Figure 1E**). A similar result was also observed in the CGGA cohort (AUC 0.9887, P < 0.001) (**Figure 1E**). These results suggested that TREM1 may serve as a biomarker for mesenchymal subtype in glioma. Then, we analyzed the intratumor distribution of TREM1 in GBM tissues by analyzing RNA sequencing data from 93 GBM samples (26). We observed that GBM tissues from the contrast-enhancing regions (GBM-CE) had higher TREM1 expression than non-enhancing (NE) margins or nonneoplastic areas (**Figure 1F**). Furthermore, based on IVY Glioblastoma Atlas Project data (*n* = 270), TREM1 was found to be enriched in pathological areas that were important for glioma progression compared to other areas, including HBV (hyperplastic blood vessels), PAN (pseudopalisading cells around necrosis), and PNZ (perinecrotic zone) (**Figure 1G**). Additionally, we found that TREM1 protein was the most strongly overexpressed protein in HGG compared with LGG and normal brain tissues from the Human Protein Atlas (**Figure 1H**). In all these above findings, it was indicated that higher TREM1 expression was accompanied by higher malignancy in glioma.

### 3.2 TREM1 predicted worse survival in glioma patients

**Figure 2:**
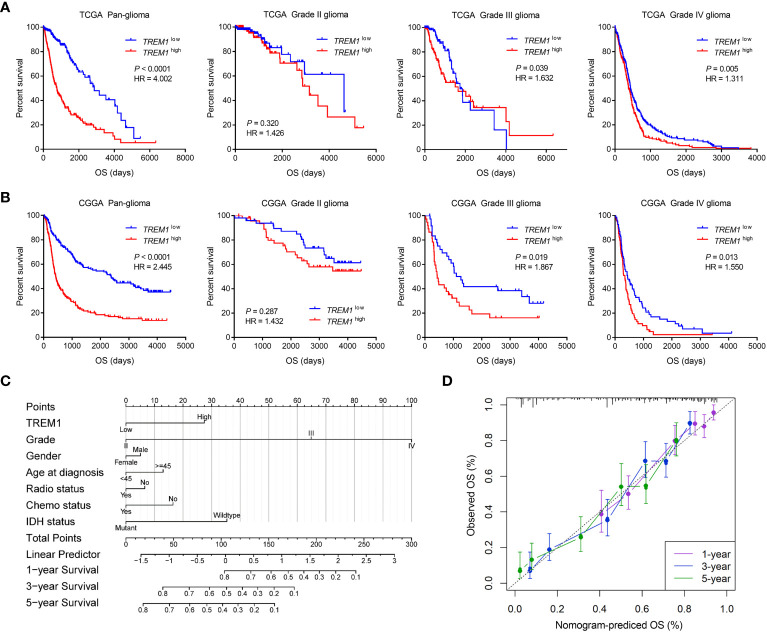
Clinical prognosis of patients with glioma with low and high TREM1 expression. **(A, B)** Kaplan-Meier analysis of overall survival (OS) based on high *vs.* low TREM1 expression in pan-glioma, grade II, grade III and grade IV patients in the TCGA and CGGA datasets. The median value of TREM1 expression was used as the cutoff value. **(C)** Nomogram for predicting the survival probability of glioma patients. **(D)** Calibration curves for predicting the 1-year, 3-year, and 5-year survival probability of glioma patients.

Kaplan-Meier analysis was further performed to investigate the prognostic value of TREM1. As shown in **Figures 2A, B**, patients with high TREM1 expression exhibited significantly shorter overall survival (OS) than their counterparts in pan-glioma analysis of TCGA and CGGA datasets. In addition, similar results were validated in the two cohorts of patients with WHO grade III and IV gliomas, although the grade II subgroup failed to reach statistical significance. Furthermore, univariate and multivariate Cox regression analysis was employed to validate TREM1 as an independent prognostic marker in glioma. Univariate Cox analysis showed that high TREM1 expression, grade, age, IDH mutation, and chemotherapy were significantly associated with overall survival in the CGGA database. Multivariate Cox regression analysis revealed that TREM1 expression was still an independent predictor for glioma patients (HR: 1.046; 95% CI: 1.019-1.117; P =0.045) after adjusting for the clinical factors mentioned above (**Supplementary Table S1**). According to the univariate Cox regression, this prognostic value significant in the TCGA dataset (**Supplementary Table S2**). A nomogram, based on statistical calculation of the risk of clinicopathological features of a cancer, has been proven to be accurate with visualization and quantification for doctors and patients, and has been widely developed to predict patient survival in the clinic (27). In this study, a nomogram model was further constructed to predict 1-year, 3-year, and 5-year survival based on the TCGA cohort (**Figure 2C**). Meanwhile, the calibration curves showed that the actual observation fit well with the predicted glioma patients’ survival probability (**Figure 2D**). All these findings collectively implied that TREM1 may serve as an independent prognostic biomarker for glioma patients.

### 3.3 TREM1 expression correlated with distinct genomic changes

**Figure 3:**
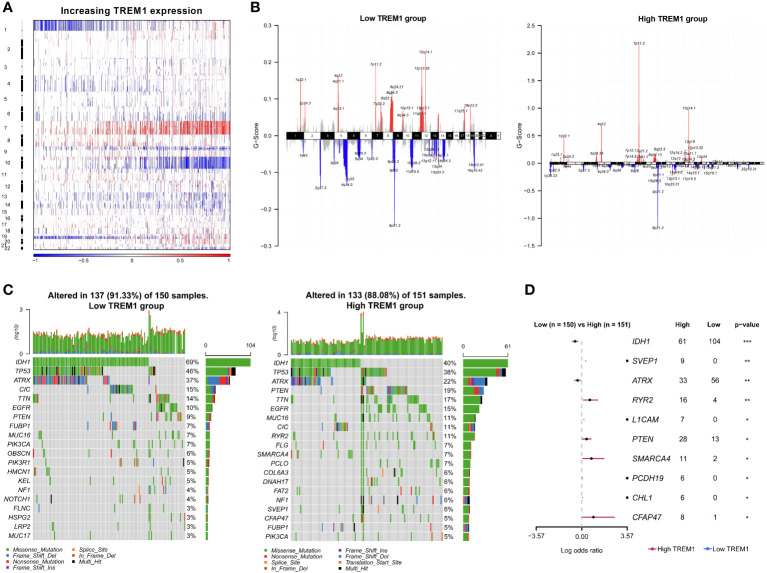
Distinct genomic changes associated with TREM1 expression. **(A)** Overview of the copy number variation (CNV) profile in order of TREM1 expression. Blue (deletion); Red (amplification). **(B)** Differential somatic mutations between low- and high-TREM1 groups. **(C)** Distinct CNV spectrum in TREM1 low and high expression groups. **(D)** Differentially mutated genes (DMGs) between the low TREM1 and high TREM1 groups. *P <0.05, **P <0.01, ***P <0.001.

To explore the molecular characteristics associated with the expression pattern of TREM1, copy number alterations and somatic mutations from TCGA database were collected. First, somatic copy number alterations were evaluated between the high and low TREM1 expression groups. As shown in **Figures 3A, B**, the incidence of 1p/19q codeletion was reduced with increasing TREM1 expression, which is a genomic characteristic of oligodendroglioma (28). Chr7 amplification accompanied by Chr10 deletion, a frequent genomic event in GBM, was enriched in the high TREM1 expression group. We analyzed all glioma cases using GISTIC 2.0 to identify 42 significantly reoccurring focal amplifications and 54 deletion events (**Supplementary Dataset S1**). In the high TREM1 expression group, focal amplification peaks, including PIK3C2B (1q32.1), PDGFRA (4q12), EGFR (7p11.2), and CDK4 (12q14.1), were well-characterized oncogenic driver genes, while this group was accompanied by a focal deletion peak in 9p21.3 (CDKN2A and CDKN2B). Additionally, somatic mutations were also analyzed based on TREM1 expression. A higher frequency of mutations in IDH1 (40%), TP53 (39%), ATRX (22%), and PTEN (19%) was observed in the high TREM1 expression cases (**Figure 3C**). As shown in **Figure 3D**, the forest plot further revealed that mutations in RYR2, SVEP1, L1CAM, PTEN, SMARCA4, PCDH19, CHL1, and CFAP47 were significantly enriched in the high TREM1 expression cases, while the low group frequently mutated in IDH1 and ATRX.

### 3.4 TREM1-related immune functions in glioma

**Figure 4:**
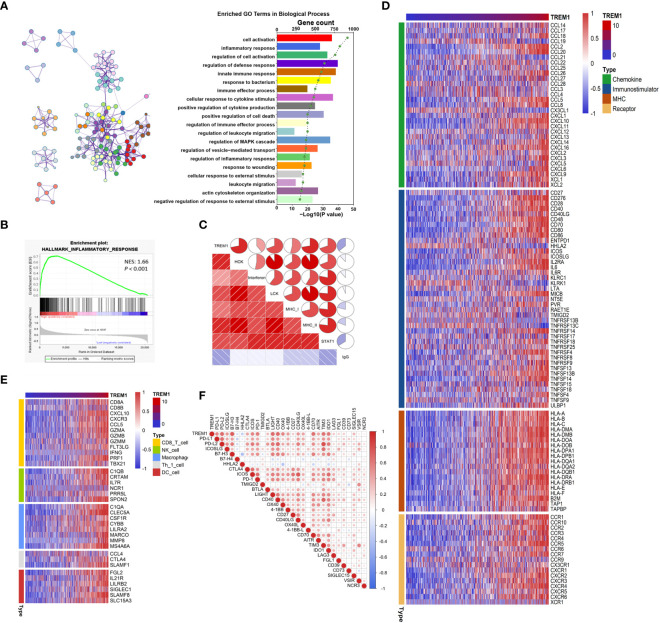
TREM1-related immune functions in glioma. **(A)** TREM1 associated with biological processes determined by GO analysis in the TCGA dataset. **(B)** GSEA-validated biological processes related to TREM1 in the TCGA dataset. NES and P value were shown for each plot. **(C)** Correlogram of TREM1 and inflammatory metagenes in the TCGA cohorts. **(D)** Correlation between TREM1 and immunomodulators (Chemokines, Immunostimulator, MHC, and Receptors) in the TCGA cohorts. **(E)** Differences in the effector genes of the tumor-associated immune cells between high- and low-TREM1 groups in the TCGA cohorts. **(F)** Correlation between TREM1 and immune checkpoints members in the TCGA cohorts.

To reveal the biological functions of glioma with different TREM1 expression levels, the genes that were strongly correlated with TREM1 expression (Pearson r > |0.5|, P <0.05) were selected in the TCGA and CGGA databases (**Supplementary Dataset S2**). Then, the related genes were explored by GO analysis in Metascape (29). GO analysis results obtained with the genes positively associated with TREM1 expression from the TCGA dataset revealed that these functions were mostly involved in inflammation and immunity, such as inflammatory response, immune response, positive regulation of cytokine production, and leukocyte migration (**Figure 4A**). The CGGA database yielded similar results as well (**Supplementary Figure 2**). GSEA was employed to validate the biological functions of TREM1, showing that the cases with high TREM1 expression had an activated phenotype for inflammatory response in the TCGA and CGGA datasets (**Figure 4B** and **Supplementary Figure 3**). In addition, based on KEGG pathology data, the majority of the positive genes associated with TREM1 expression were involved in immune-related pathways (**Supplementary Figure 4A**). The signaling network according to the results from KEGG pathway analysis suggested an association between TREM1 and inflammatory-immune related pathways, such as NF-κB signaling, TNF signaling, Toll-like receptor signaling, and chemokine signaling pathways (**Supplementary Figure 4B**).

Based on the results mentioned above, we explored the role of TREM1 in inflammation using seven metagenes as previously described (30). In both the TCGA and CGGA datasets, TREM1 expression was found to be positively correlated with HCK, interferon, LCK, MHC-I, MHC-II, and STAT1 metagenes but negatively correlated with IgG metagenes in glioma (**Figure 4C** and **Supplementary Figure 5**). Besides, multiple immune modulators were observed to be strong positive associated with TREM1 expression level, including some key monocyte/macrophage chemokines (CCL2, CCL22, *etc.*) and MHC molecules (**Figure 4D** and **Supplementary Figure 6**). We also observed that TREM1 was positively correlated with marker genes of tumor infiltrating cells, such as CD8^+^ T cell, NK cell, DC cell, and macrophage (**Figure 4E** and **Supplementary Figure 7**).

Immune checkpoints are extremely important molecules in the regulation of immune processes. We next examined the correlationship between TREM1 and immune checkpoints expression. We enrolled well-known immune checkpoints in this analysis, including the B7-CD28 family (PD-L1, PD-L2, ICOSLG, B7-H3, B7-H4, HHLA2, CTLA4, ICOS, PD-1, and TMIGD2), TNF superfamily (BTLA, LIGHT, CD40, OX40, 4-1BB, CD27, CD40LG, OX40L, 4-1BB-L, CD70, and AITR), and other immune checkpoint members (TIM3, IDO1, LAG3, FGL1, CD39, CD73, SIGLEC15, VSIR, and NCR3) (31). It was found that TREM1 expression was positively correlated with most of these immune checkpoint molecules in both the CGGA and TCGA cohorts (**Figure 4F** and **Supplementary Figure 8**). Moreover, the TIDE-score was used to assess the potential clinical efficacy of immunotherapy in different risk groups (32). In our results, TREM1-high group had a higher TIDE score than the low group in the CGGA database. Also, TREM1-high group had a lower microsatellite instability (MSI) score, and higher T cell dysfunction score, but there was no difference in T cell exclusion between the two groups (**Supplementary Figure 9**). It was suggested that patients with low TREM1 could benefit more from ICI therapy. TREM1 might be a predictive marker for glioma immunotherapy. Taking these findings together, TREM1 may play an important role in immunobiologic processes of glioma.

### 3.5 Association of TREM1 expression with immune cell populations in the glioma microenvironment

**Figure 5:**
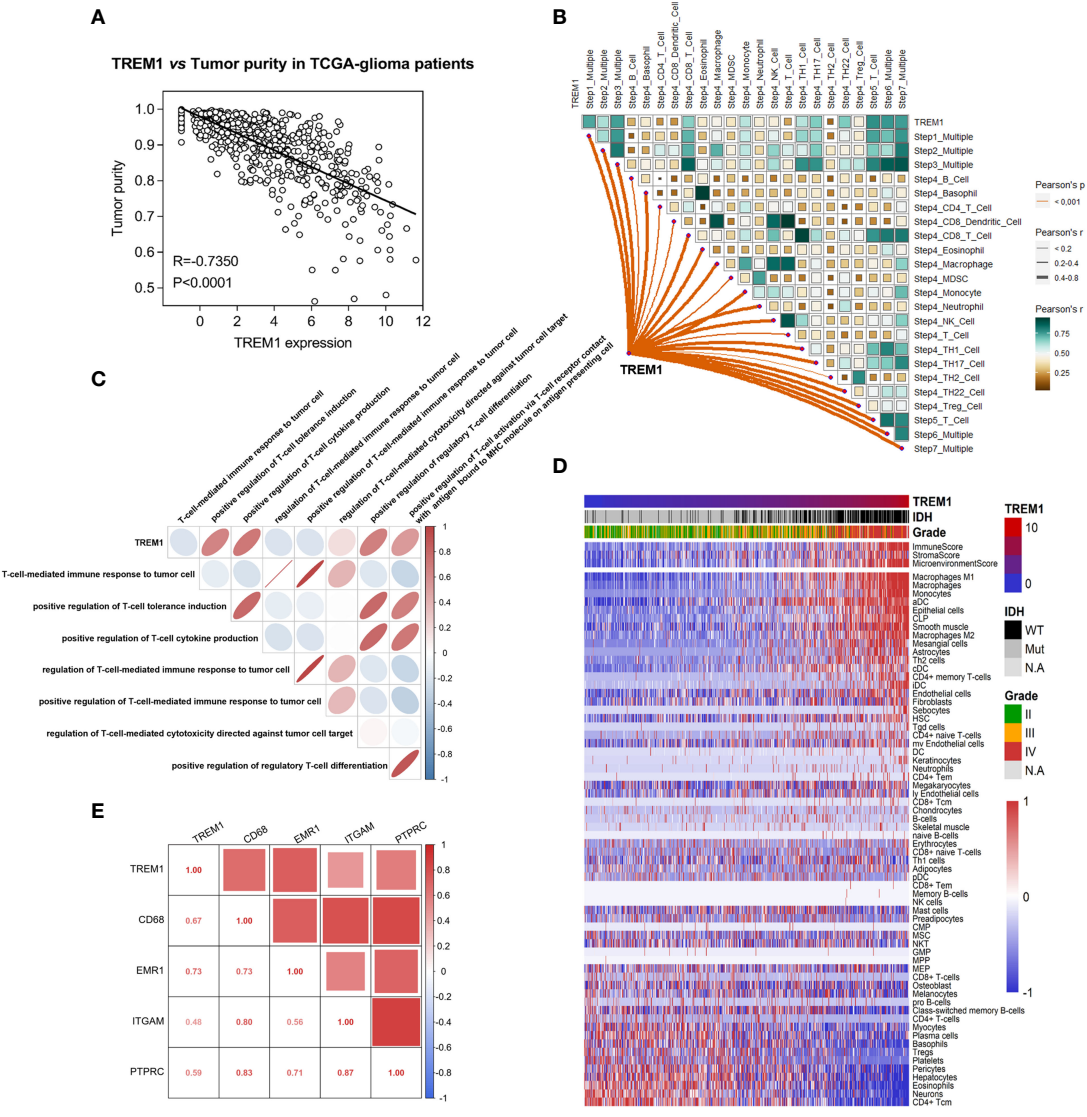
Association of TREM1 expression with immune cell populations in the glioma microenvironment. **(A)** Correlation analysis between TREM1 expression levels and tumor purity in the TCGA database. **(B)** Correlation between TREM1 and the steps of the cancer immunity cycle in the TCGA database. **(C)** Relationship between TREM1 expression levels and T-cell-specific immunity in the TCGA database. **(D)** Heatmaps showing the relationship between TREM1 expression and immune and stromal cell populations in the TCGA database. Expression values were subjected to z-score normalization, with high levels shown in red and low levels shown in blue. **(E)** Correlation analysis between TREM1 and macrophage marker genes in the TCGA database.

Just like other cancers, glioma tissues are also composed of not only neoplastic cells but also nonneoplastic cells, such as stromal cells and immune cells. It was reported that these nonneoplastic cells diluted the purity of glioma and played important roles in the progression of glioma (4). For a thorough understanding of the relationship between neoplastic and nonneoplastic cells, the ESTIMATE algorithm method was designed (33). We found that TREM1 expression was negatively correlated with tumor purity (TCGA-seq: R= -0.7350, P <0.0001; CGGA-seq: R= -0.7126, P <0.0001) in the TCGA and CGGA datasets (**Figure 5A** and **Supplementary Figure 10A**).

In the areas of cancer immunology and immunotherapy, the cancer immunity cycle has become the intellectual framework (34). A majority of steps in the cancer immunity cycle were up-regulated in the high-TREM1 subgroup, including the release of cancer cell antigens (Step 1), priming and activation (Step 3), and trafficking of immune cells to tumors (Step 4) (CD8^+^ T cell recruiting, macrophage recruiting, and Th1 cell recruiting), infiltration of immune cells into tumors (Step 5), recognition of cancer cells by T cells (Step 6), and killing of cancer cells (Step 7) (**Figure 5B** and **Supplementary Figure 10B**). Moreover, GSVA analysis was found that TREM1 was positively correlated with positive regulation of T cell tolerance induction (GO:0002666), positive regulation of T cell cytokine production (GO:0002726), positive regulation of regulatory T cell differentiation (GO:0045591), and positive regulation of T cell activation via T cell receptor contact with antigen bound to MHC molecule on antigen presenting cell (GO:2001190) in both of the TCGA and CGGA cohorts (**Figure 5C** and **Supplementary Figure 10C**).

In addition, the relationship between TREM1 and nontumor cells in the tumor microenvironment was determined via TIMER2.0 (http://timer.cistrome.org/). We found that TREM1 positively correlated with the infiltration levels of the immune cells, including monocytes and macrophages, across human cancers (**Supplementary Figure 11**). And the relationship between TREM1 and 64 immune and stromal cell types was determined using the xCell method (35). As shown in **Figure 5D** and **Supplementary Figure 10D**, TREM1 positively correlated with multiple infiltrating immune cell types, especially monocytes and macrophages. Further, TREM1 was positively correlated with marker genes of infiltrating macrophages cells (**Figure 5E** and **Supplementary Figure 10E**). The above findings suggested that TREM1 was closely associated with monocytes/macrophages to contribute to the generation of an immunosuppressive tumor microenvironment.

### 3.6 TREM1 was associated with chemotaxis and cytokine secretion of monocytes

**Figure 6:**
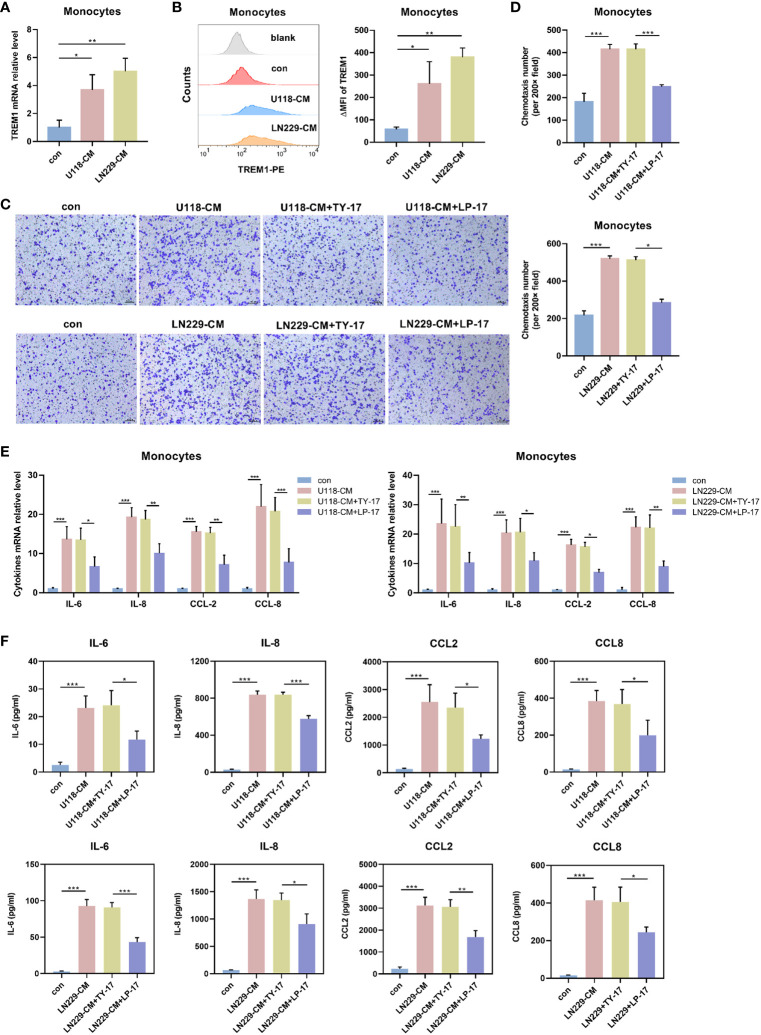
TREM1 regulated chemotaxis and cytokine secretion profile of monocytes. **(A, B)** TREM1 expression in CD14^+^ monocytes was induced by U118-CM/LN229-CM for 48 h. The mRNA levels of TREM1 were determined by qRT-PCR **(A)** and TREM1 protein was detected using flow cytometry **(B)**. **(C, D)** After pretreatment with U118-CM/LN229-CM in the presence or absence of TY-17 at 200 ng/ml or LP-17 at 200 ng/ml for 48 h, typical images **(C)** and quantification **(D)** of migrated primary CD14^+^ monocytes *in vitro* (×200). **(E, F)** CD14^+^ monocytes were incubated with U118-CM/LN229-CM containing TY-17 or LP-17 for 48 h, cytokine transcription **(E)** and concentration **(F)** in CM were determined by qRT-PCR and ELISA, respectively. Each bar represents the mean ± SD (n = 3). *P < 0.05; **P < 0.01; ***P < 0.001.

Considering the substantial link between TREM1 and monocytes, we tested whether glioma cells can regulate the expression of TREM1 on monocytes. We found that U118/LN229-CM significantly increased the mRNA and protein levels of TREM1 in primary CD14^+^ circulating monocytes after 48 h of treatment (**Figures 6A, B**). We investigated the role of TREM1 in the recruitment of monocytes to tumors. Transwell assays were used to evaluate chemotaxis of CD14^+^ monocytes towards the CM of glioma cells. After pretreatment with U118-CM/LN229-CM in the presence of LP-17 at 200 ng/ml for 48 h, the chemotaxis of primary monocytes towards glioma CM was significantly inhibited (**Figures 6C, D**). We further examined whether increased TREM1 expression was also associated with cytokine secretion by monocytes. After pretreatment with glioma CM containing LP-17 at 200 ng/ml for 48 h, monocytes were washed and cultured in fresh medium for another 24 h. The mRNA and secretion levels of IL-6, IL-8, CCL2, and CCL8 in monocytes increased significantly after U118-CM/LN229-CM stimulation, whereas their upregulation was offset by TREM1 inhibitor (**Figures 6E, F**). Together, these results indicated that TREM1 was associated with chemotaxis and cytokine secretion of monocytes.

### 3.7 TREM1 was associated with biological functions of glioma-educated macrophages

**Figure 7:**
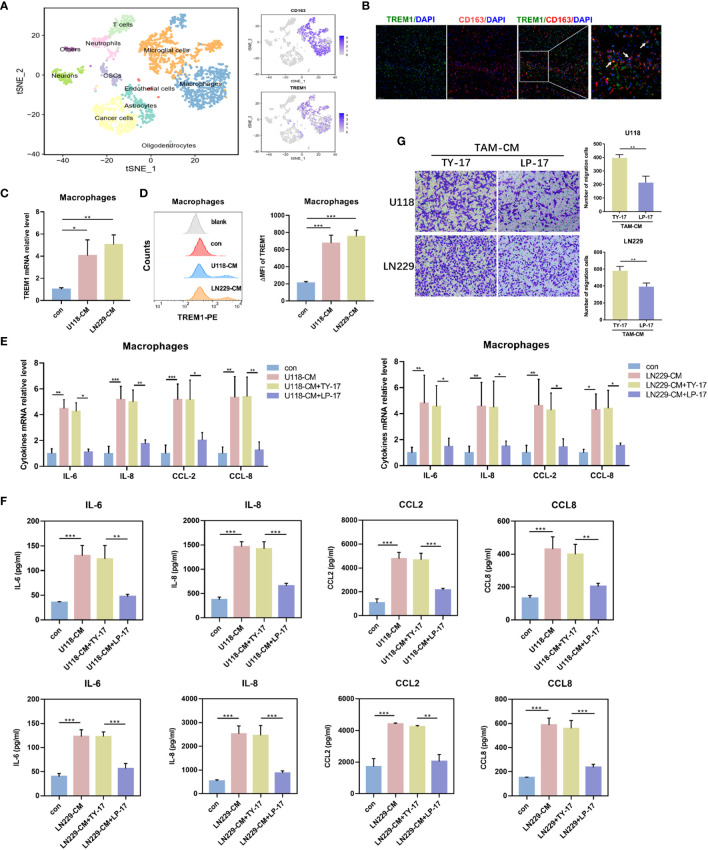
TREM1 regulated biological functions of glioma-educated macrophages. **(A)** tSNE plot showed different cell clusters of GBM samples from the GSE84465 dataset. **(B)** The representative photos of polychromatic immunohistochemical staining of TREM1, CD163 and DAPI in glioma tissues (green: TREM1; red: CD163; blue: cell nuclei). **(C, D)** The mRNA levels of TREM1 in TAMs were determined by qRT-PCR **(C)** and TREM1 protein were detected using Flow cytometry **(D)**. **(E, F)** TAMs were incubated with TY-17 or LP-17 for 48 h, and then were washed with PBS and further cultured in fresh DMEM medium for 24 h. Cytokines transcription **(E)** and concentration **(F)** in the medium were determined by qRT-PCR and ELISA, respectively. **(G)** Transwell assay to analyze the effect of blocking TREM1 in TAMs on glioma cell migration (×100). Each bar represents the mean ± SD (n = 3). *P<0.05; **P < 0.01; ***P < 0.001. TAM-CM, conditioned medium from monocyte-derived TAMs.

As showed in **Figure 5D**, human tumor-infiltrated macrophages showed relatively link with TREM1. To validate this hypothesis, we conducted single-cell sequencing analysis GBM samples from GSE84465 dataset. tSNE plot showed that macrophages expressed TREM1 (**Figure 7A**). Moreover, polychromatic immunohistochemical staining demonstrated that TREM1 was co-localized with the surface markers of macrophages CD163 (**Figure 7B**). In line with this, we generated TAMs from CD14^+^ monocytes in a 7-day-long differentiation process, using U118-CM/LN229-CM. We observed increased mRNA and protein levels of TREM1 on the TAMs (**Figures 7C, D**). We further analyzed the secretion profiles of TAMs. Compared with control-derived macrophages, glioma-derived TAMs produced higher levels of IL-6, IL-8, CCL2, and CCL8. After blocking TREM1, glioma-derived TAMs expressed less levels of IL-6, IL-8, CCL2 and CCL8 (**Figures 7E, F**). Glioma-derived TAMs were incubated with TY-17 or LP-17 for 48 h, then washed with PBS and further cultured in fresh DMEM medium for 24 h (TY17-TAM-CM or LP17-TAM-CM). We found glioma cells that co-cultured with LP17-TAM-CM exhibited reduced migratory capacity compared with control group (**Figure 7G**). These results indicated that TREM1 reshaped the microenvironment through regulating macrophage cytokine secretion and altered the glioma migration through TAMs.

## 4 Discussion

The management of advanced-stage malignancies has been greatly transformed by the development of cancer immunotherapy. Immune checkpoint blockade therapy has revolutionized the treatment of several tumor subtypes (36). However, the antitumor effect of monotherapy with immune checkpoint inhibitory agents is limited to certain types of cancer, such as glioma, with an effective response rate of less than 30% (36, 37). It is these unfavorable results that encourage us to identify other immune targets and optimize treatment strategies.

TREM1 has recently been identified as a new potential target for immunotherapy. It has been demonstrated that TREM1 inhibitors can attenuate tumor growth and promote the antitumor efficacy of blocking PD-L1 (21). However, to date, there has been no systematic research on TREM1 in glioma. In this study, a large-scale, comprehensive, bioinformatic analysis was used to characterize the landscape of TREM1 among glioma. We observed that TREM1 expression increased with the grade of malignancy in glioma. TREM1 was enriched in the mesenchymal subtype and significantly upregulated in IDH wild-type glioma. TREM1 was localized to perinecrotic regions, pseudopalisading cells around necrosis, and hyperplastic blood vessels. In addition, our study suggested higher TREM1 expression levels predicted worse survival among patients with glioma, especially GBM. Importantly, TREM1 was a valuable independent prognostic factor in glioma. There appears to be an association between TREM1 and a malignant phenotype and that it may be involved in glioma progression.

We then conducted GO and KEGG analysis to understand the main biological processes and pathways associated with TREM1 in whole glioma. Our analysis showed that TREM1 was a critical player in regulating immune-related pathways, such as NF-κB and TNF signaling, which were essential for controlling the transcription of key genes. TREM1 was also associated with multiple immune modulators, especially some MHC molecules and chemokines. These chemokines could recruit immune cells, including monocytes and macrophages, to create an immunosuppressive microenvironment, thereby undermining the efficacy of immunotherapies. Clinical trials of immune checkpoint blockades for glioma are ongoing (36, 37). Following that, we investigated the relationship between TREM1 and immune checkpoint molecules. TREM1 was strongly synergistic with most of known immune checkpoint molecules, such as B7-H3, B7-H4, PD-L1, TIM3, and PD-1, in two independent public databases. Evidence has revealed that TREM1 inhibitors can sensitize tumor cells to anti-PD-L1 immunotherapy for liver cancer (21). In addition, one clinical trial has already reported that dual PD-1 and CSF-1R blockade may provide anti-tumor effects which are durable for malignant glioma patients (ClinicalTrials.gov identifier: NCT02526017). Therefore, combination therapy will be the mainstream treatment for glioma in the future (38, 39). Although TREM1 inhibitors are currently unavailable for clinical application, a desirable relationship between TREM1 and other immune checkpoints maps out a rosy blueprint for the combination therapy of glioma.

Studies have revealed the importance of tumor purity in clinical features and identified the resulting enriched immune cells. Low purity indicates gliomagenesis, malignancy progression, and an enhanced immune phenotype (36). Our results showed that TREM1 was just closely associated with low purity. We observed a positive correlation between the TREM1-high subgroup and immune activation, while also noting an immunosuppressive state. This characteristic elucidated the underlying reason for the enrichment of immune activation in the TREM1-high subgroup, without hindering glioma progression. Previous studies have reported that TREM1 is expressed on immune cells (40). Similarly, we found that high TREM1 was accompanied by high levels of immune infiltration, including monocytes and macrophages. *In vitro* biological and functional assays showed that increased expression of TREM1 enhanced chemotaxis of monocytes and altered TAM composition in the TME of glioma. In summary, these data suggested TREM1 function as a contributor to remodeling the immunosuppressive microenvironment and promoting malignant progression of glioma.

Despite these findings, there are still some existing limitations in our study. Firstly, it is necessary to conduct further studies with appropriate glioma immunotherapy cohorts to validate the predictive value of TREM1 for glioma immunotherapy response, and analysis with more detailed clinical information should be presented in the following research. Furthermore, we did not systematically investigate the detailed mechanisms of TREM1 regulating the immune microenvironment and promoting the efficacy of immunotherapy in glioma, which we will focus on studying in future studies. We could conduct functional verification experiments using patient-derived glioma cells, which had better representation of tumor heterogeneity, retention of original genomic and epigenomic features. Glioblastoma patient-derived orthotopic xenografts should also be employed to verify that blocking TREM1 could decrease tumor-promoting effects of monocytes/TAMs. In conclusion, our study illustrated that TREM1 as a potential independent prognostic factor and immune target that may improve clinical efficacy in glioma treatment.

## Data availability statement

The datasets presented in this study can be found in online repositories. The names of the repository/repositories and accession number(s) can be found in the article/**Supplementary Material**.

## Ethics statement

The studies involving humans were approved by the Ethics Committee of Qilu Hospital of Shandong University (KYLL-202111-083-1). The studies were conducted in accordance with the local legislation and institutional requirements. The human samples used in this study were acquired from primarily isolated as part of your previous study for which ethical approval was obtained. The participants provided their written informed consent to participate in this study.

## Author contributions

LZ: Conceptualization, Data curation, Methodology, Software, Writing – original draft. XQ: Investigation, Visualization, Writing – review & editing. YX: Investigation, Visualization, Writing – review & editing, Conceptualization, Data curation, Methodology.

## Supplementary material

Supplementary Figure 1 **(A)** Analysis of TREM1 mRNA levels in WHO grade II-IV gliomas from the CGGA microarray and GSE16011datasets. **(B)** Analysis of TREM1 mRNA levels in the histopathologic classification from the GSE16011 (*n* = 284) and CGGA (RNA-seq, *n* = 693) datasets. **(C)** TREM1 expression in IDH1 mutant status groups from the TCGA (RNA-seq, *n* = 667) and CGGA (RNA-seq, *n* = 693, microarray, *n* = 301) datasets.

Supplementary Figure 2 TREM1 associated with biological processes determined with GO analysis in the CGGA dataset.

Supplementary Figure 3 GSEA-validated biological processes related to TREM1 in the CGGA dataset. NES and P value were shown for each plot.

Supplementary Figure 4 **(A)** KEGG pathway analysis of TREM1 positively related genes in the TCGA and CGGA datasets. **(B)** Network construction of pathways enriched in TREM1-positively associated gene signatures in the TCGA and CGGA cohorts.

Supplementary Figure 5 Correlogram of TREM1 and inflammatory metagenes in the CGGA cohorts.

Supplementary Figure 6 Correlation between TREM1 and immunomodulators (Chemokines, Immunostimulator, MHC, and Receptors) in the CGGA cohorts.

Supplementary Figure 7 Differences in the effector genes of the tumor-associated immune cells between high- and low-TREM1 groups in the CGGA cohorts.

Supplementary Figure 8 Correlation between TREM1 and immune checkpoints members in the CGGA cohorts.

Supplementary Figure 9 TIDE score, MSI, T cell Dysfunction and Exclusion in high- and low-TREM1 groups in the CGGA cohorts. The score between the two groups were compared through Wilcoxon test (ns, not significant; ***P < 0.001)

Supplementary Figure 10 **(A)** Correlation analysis between TREM1 expression levels and tumor purity in the CGGA database. **(B)** Correlation between TREM1 and the steps of the cancer immunity cycle in the CGGA database. **(C)** Relationship between TREM expression levels and T-cell-specific immunity in the CGGA database. **(D)** Heatmaps showing the relationship between TREM1 expression and immune and stromal cell populations in the TCGA database. Expression values were subjected to z-score normalization, with high levels shown in red and low levels shown in blue. **(E)** Correlation analysis between TREM1 and macrophage marker genes in the CGGA database.

Supplementary Figure 11 Correlation analysis between TREM1 and nontumor cells in the tumor microenvironment across human cancers in the TCGA database.
