# Supplementary figures and images for: Molecular and immunological features of TREM1 and its emergence as a prognostic indicator in glioma
Source: Front Immunol. 2024 Feb 2;15:1324010. doi: 10.3389/fimmu.2024.1324010 (PMC10869492; doi:10.3389/fimmu.2024.1324010)

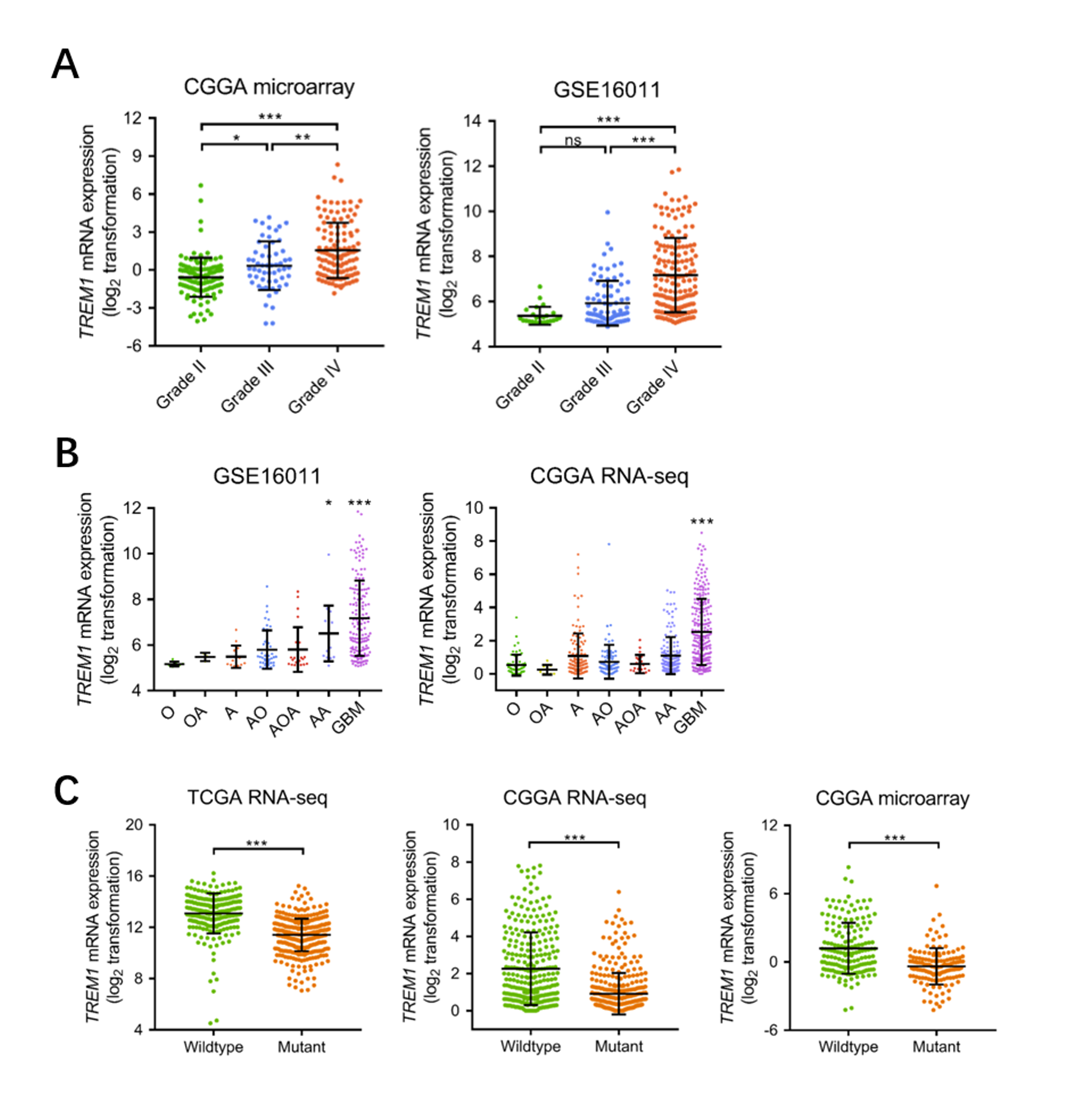

Supplement: Supplementary Figure 1 — (A) Analysis of TREM1 mRNA levels in WHO grade II-IV gliomas from the CGGA microarray and GSE16011datasets. (B) Analysis of TREM1 mRNA levels in the histopathologic classification from the GSE16011 (n = 284) and CGGA (RNA-seq, n = 693) datasets. (C) TREM1 expression in IDH1 mutant status groups from the TCGA (RNA-seq, n = 667) and CGGA (RNA-seq, n = 693, microarray, n = 301) datasets. [file Image_1.tif]

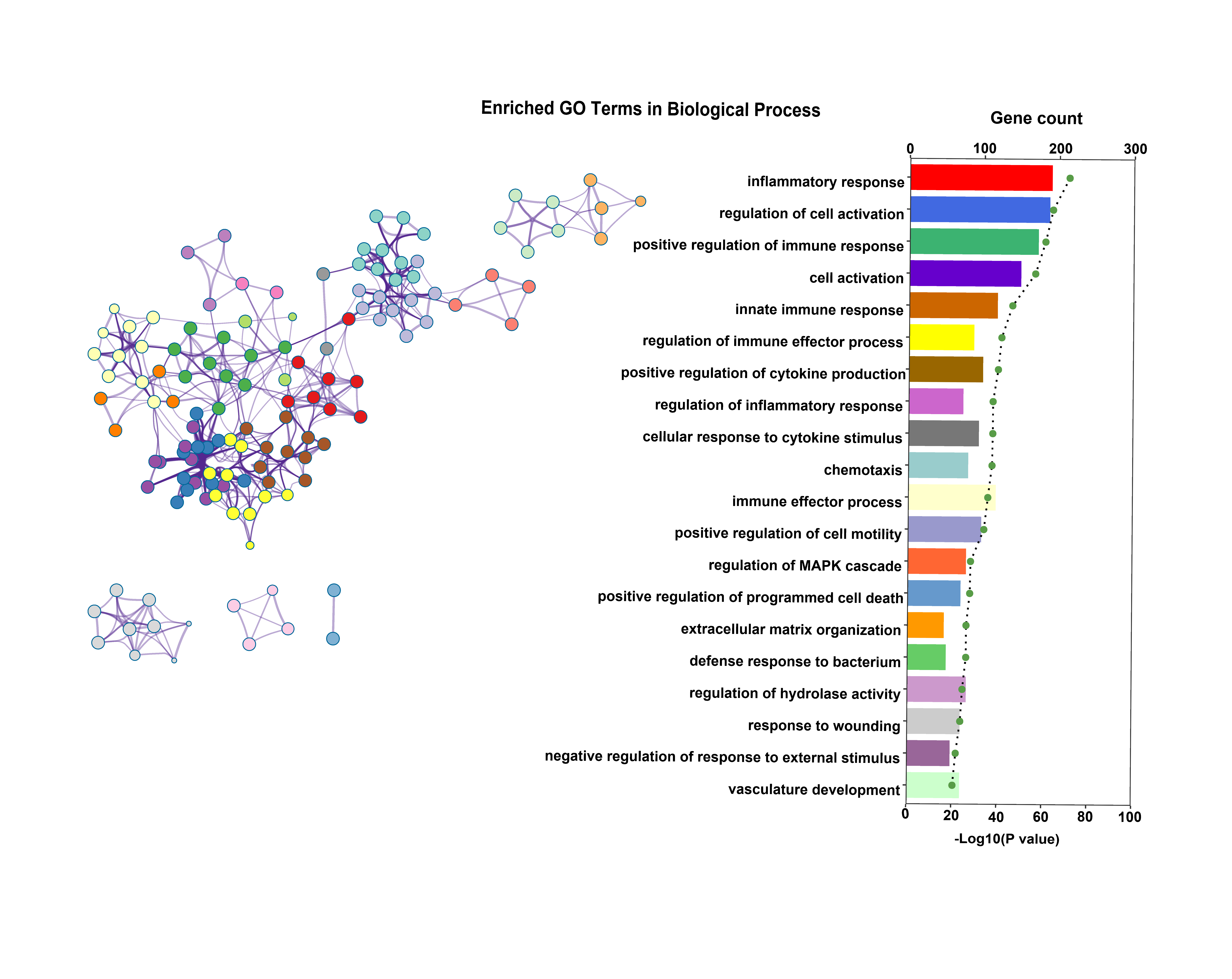

Supplement: Supplementary Figure 2 — TREM1 associated with biological processes determined with GO analysis in the CGGA dataset. [file Image_2.tiff]

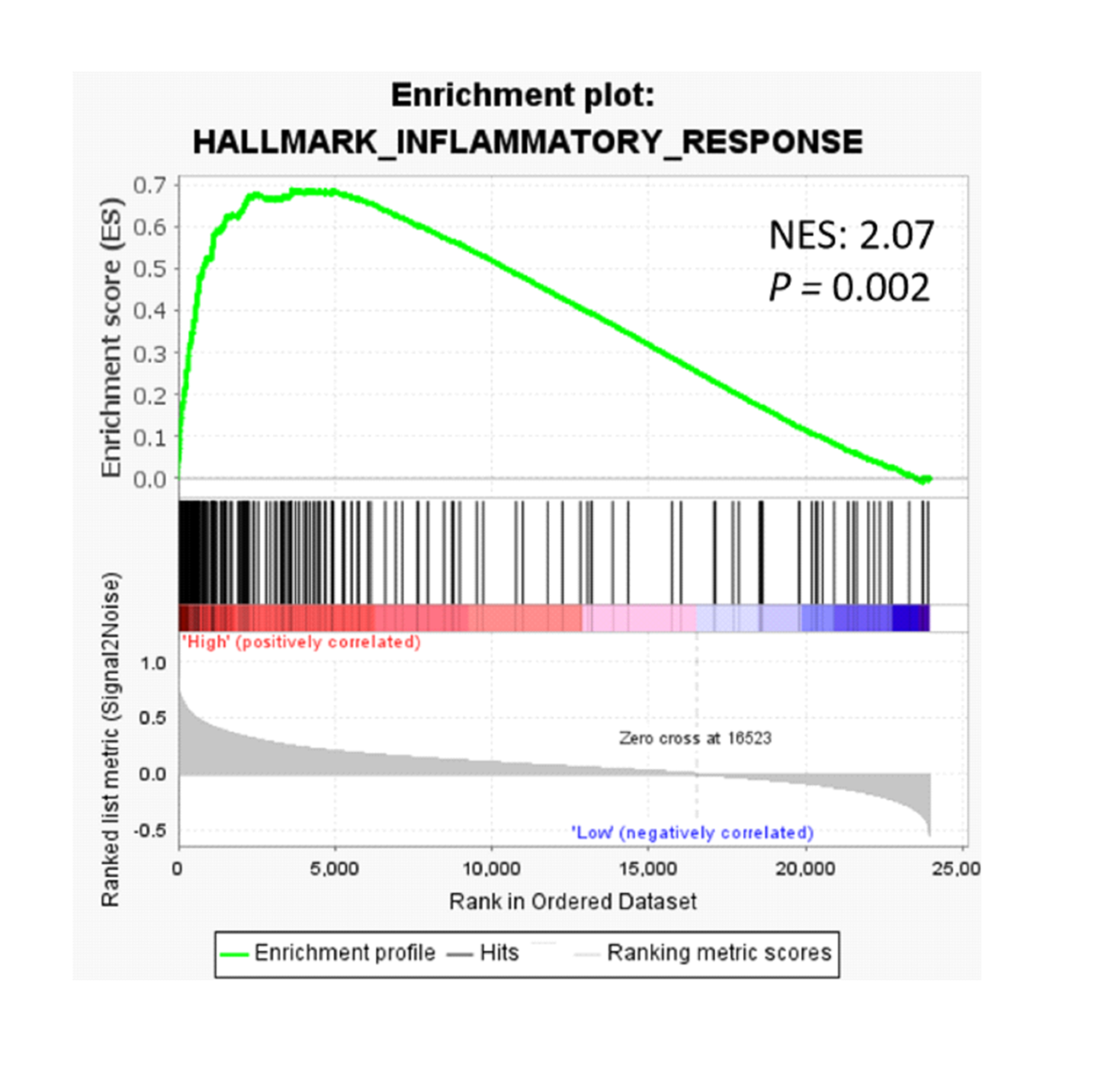

Supplement: Supplementary Figure 3 — GSEA-validated biological processes related to TREM1 in the CGGA dataset. NES and P value were shown for each plot. [file Image_3.tif]

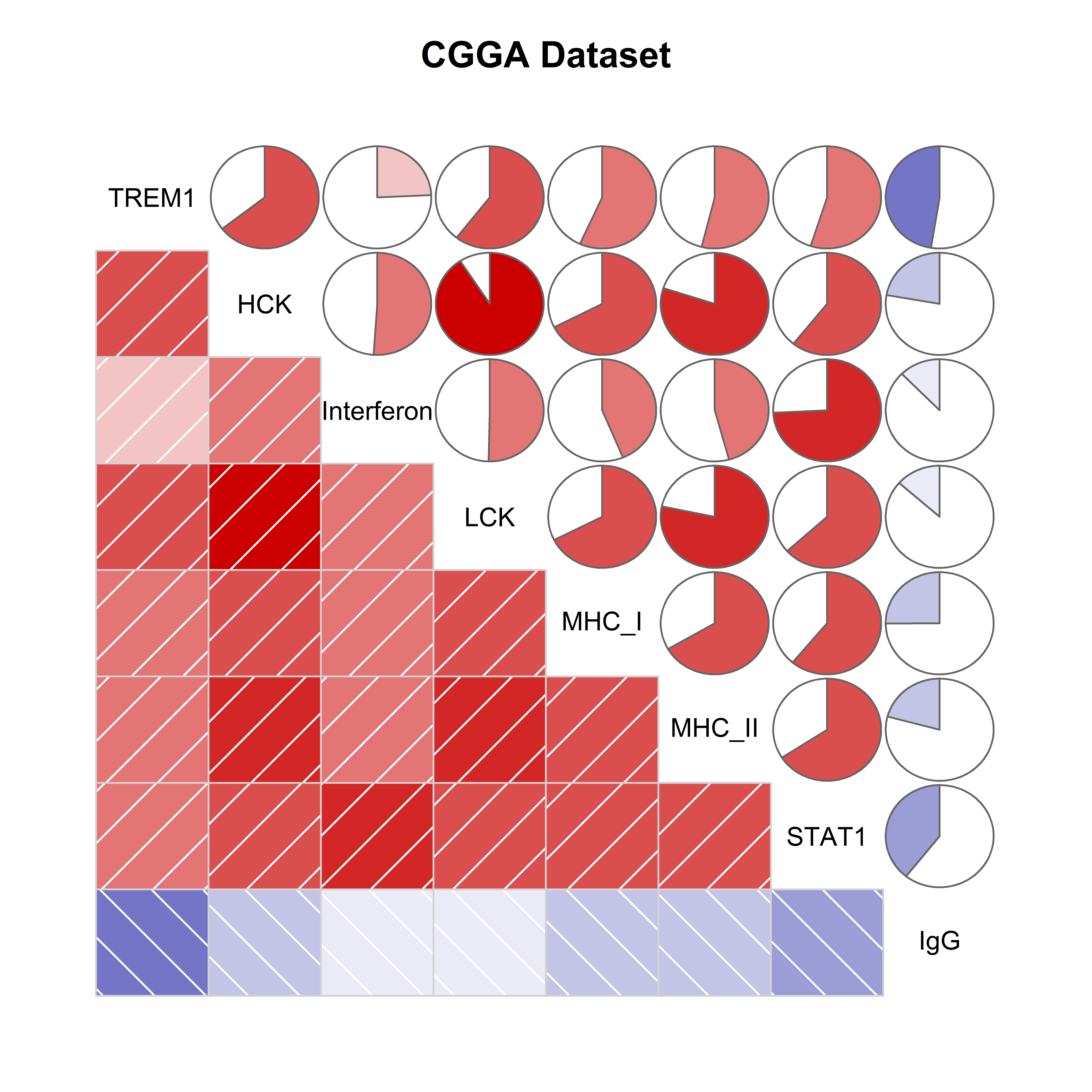

Supplement: Supplementary Figure 5 — Correlogram of TREM1 and inflammatory metagenes in the CGGA cohorts. [file Image_5.tiff]

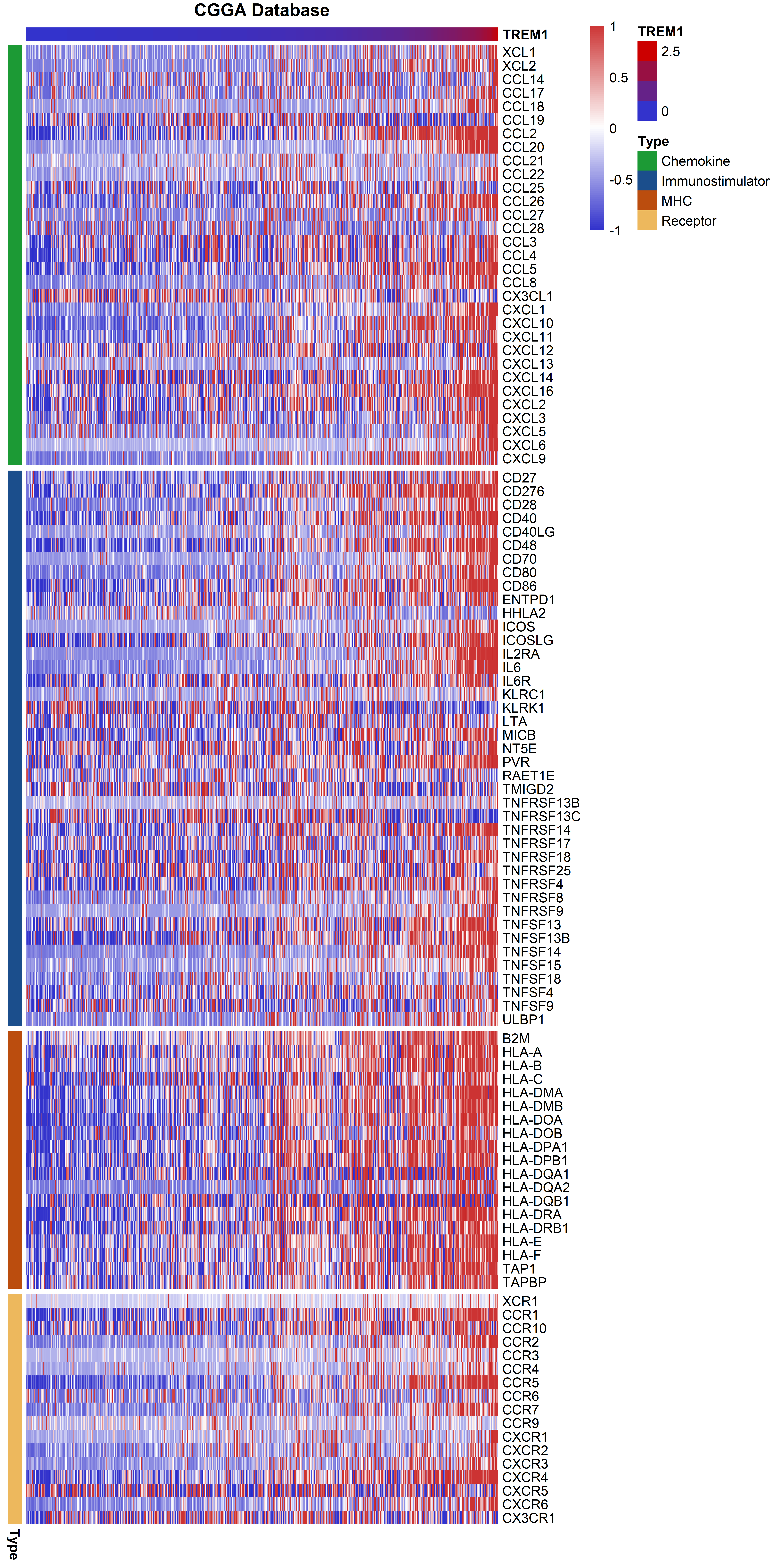

Supplement: Supplementary Figure 6 — Correlation between TREM1 and immunomodulators (Chemokines, Immunostimulator, MHC, and Receptors) in the CGGA cohorts. [file Image_6.tiff]

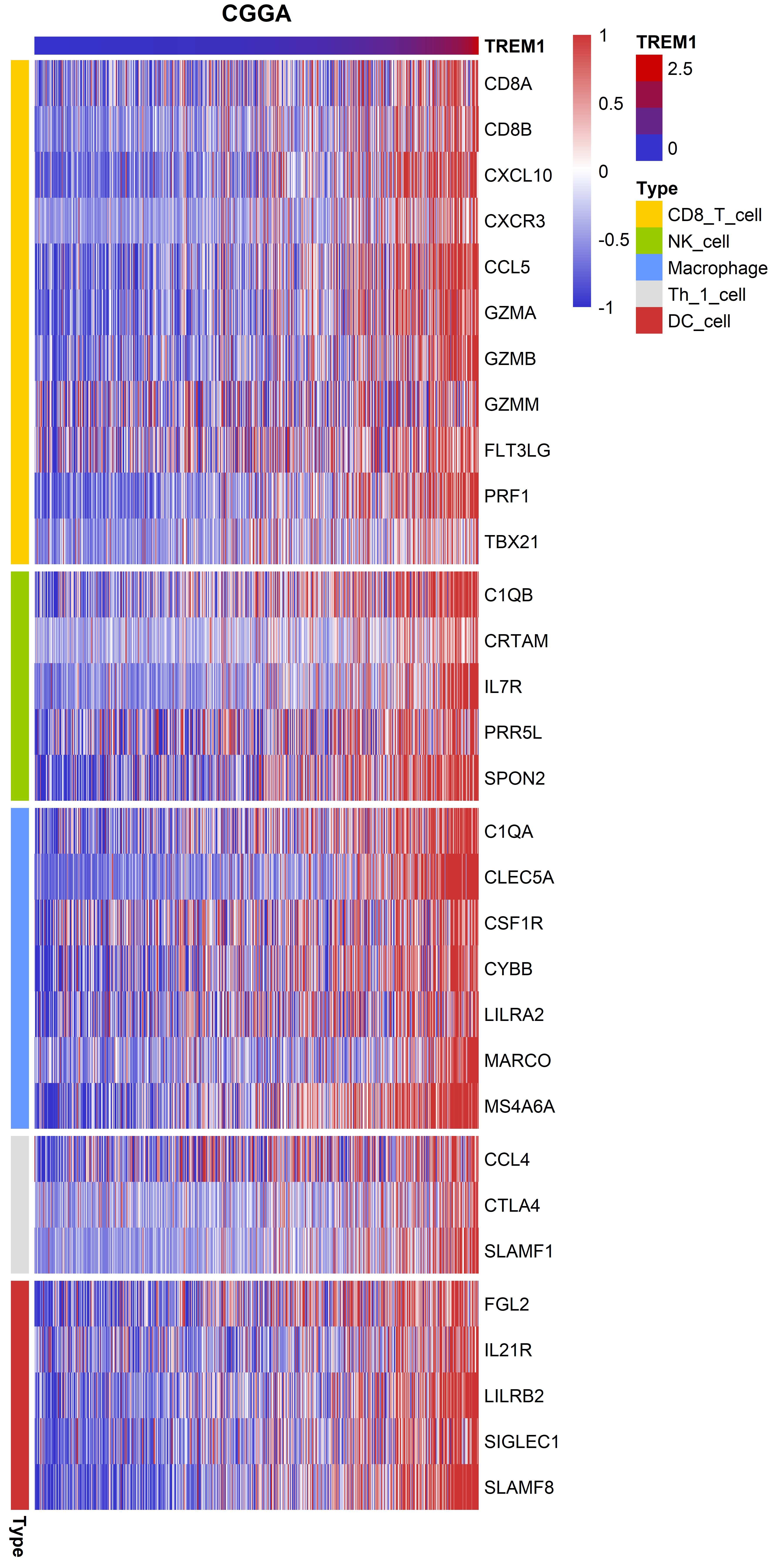

Supplement: Supplementary Figure 7 — Differences in the effector genes of the tumor-associated immune cells between high- and low-TREM1 groups in the CGGA cohorts. [file Image_7.tiff]

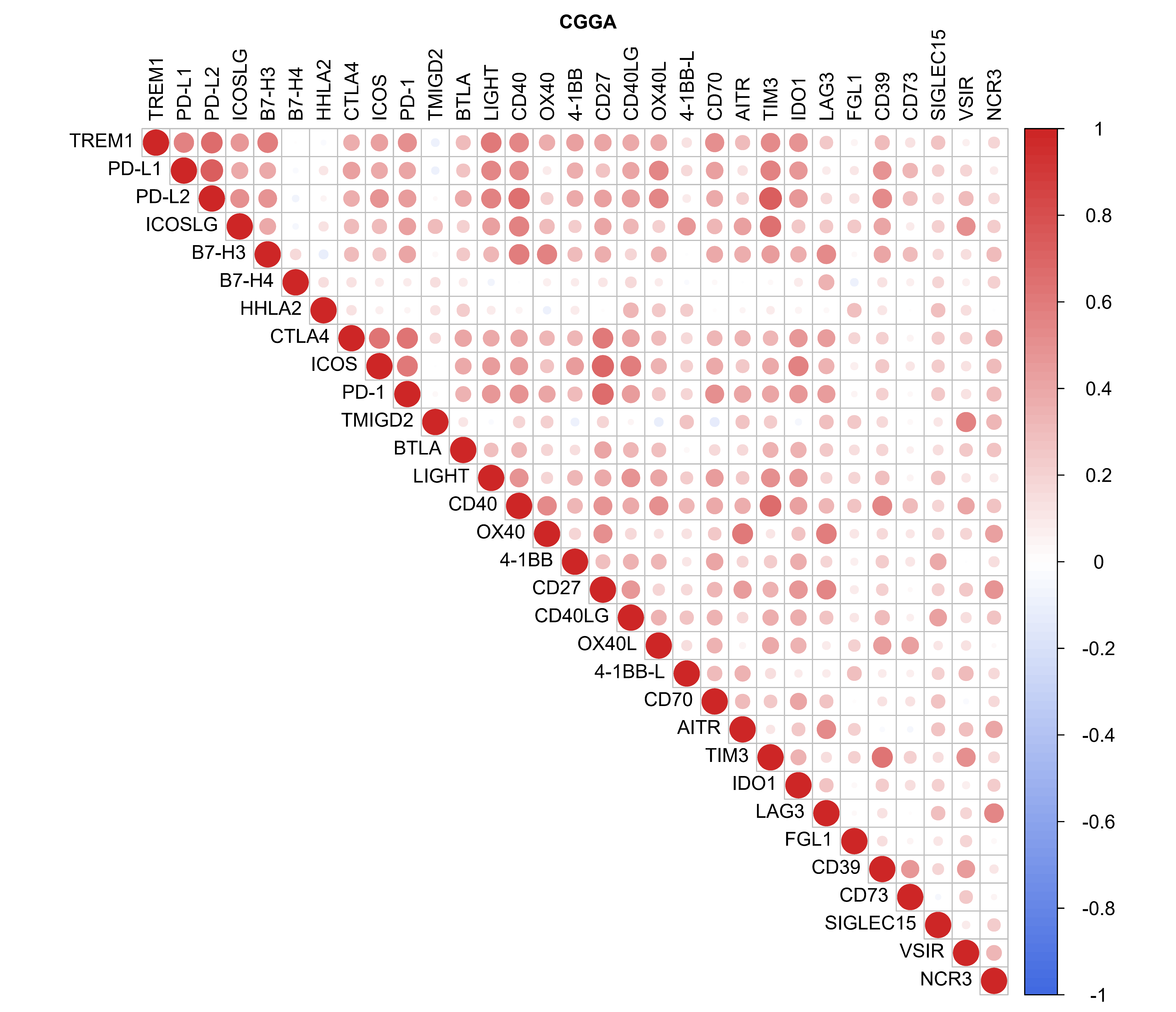

Supplement: Supplementary Figure 8 — Correlation between TREM1 and immune checkpoints members in the CGGA cohorts. [file Image_8.tiff]

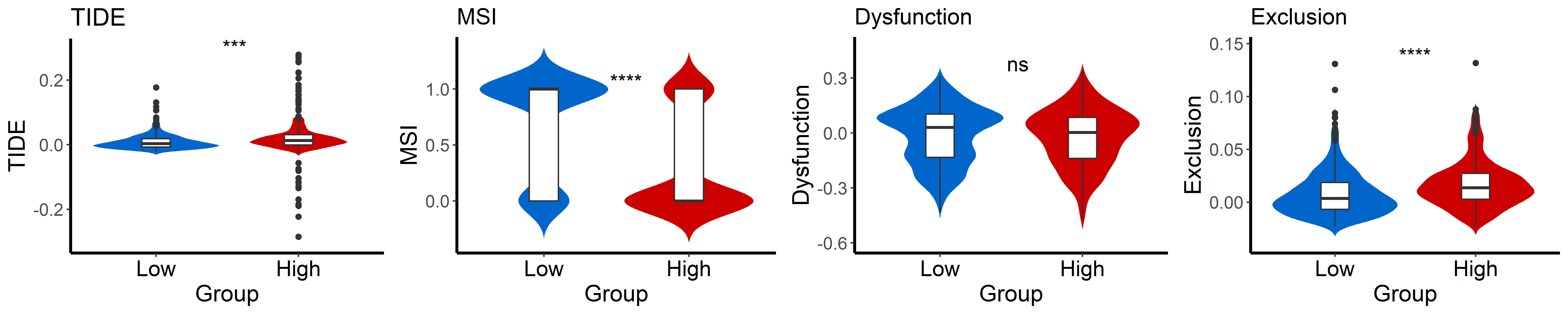

Supplement: Supplementary Figure 9 — TIDE score, MSI, T cell Dysfunction and Exclusion in high- and low-TREM1 groups in the CGGA cohorts. The score between the two groups were compared through Wilcoxon test (ns, not significant; ***P < 0.001) [file Image_9.tiff]

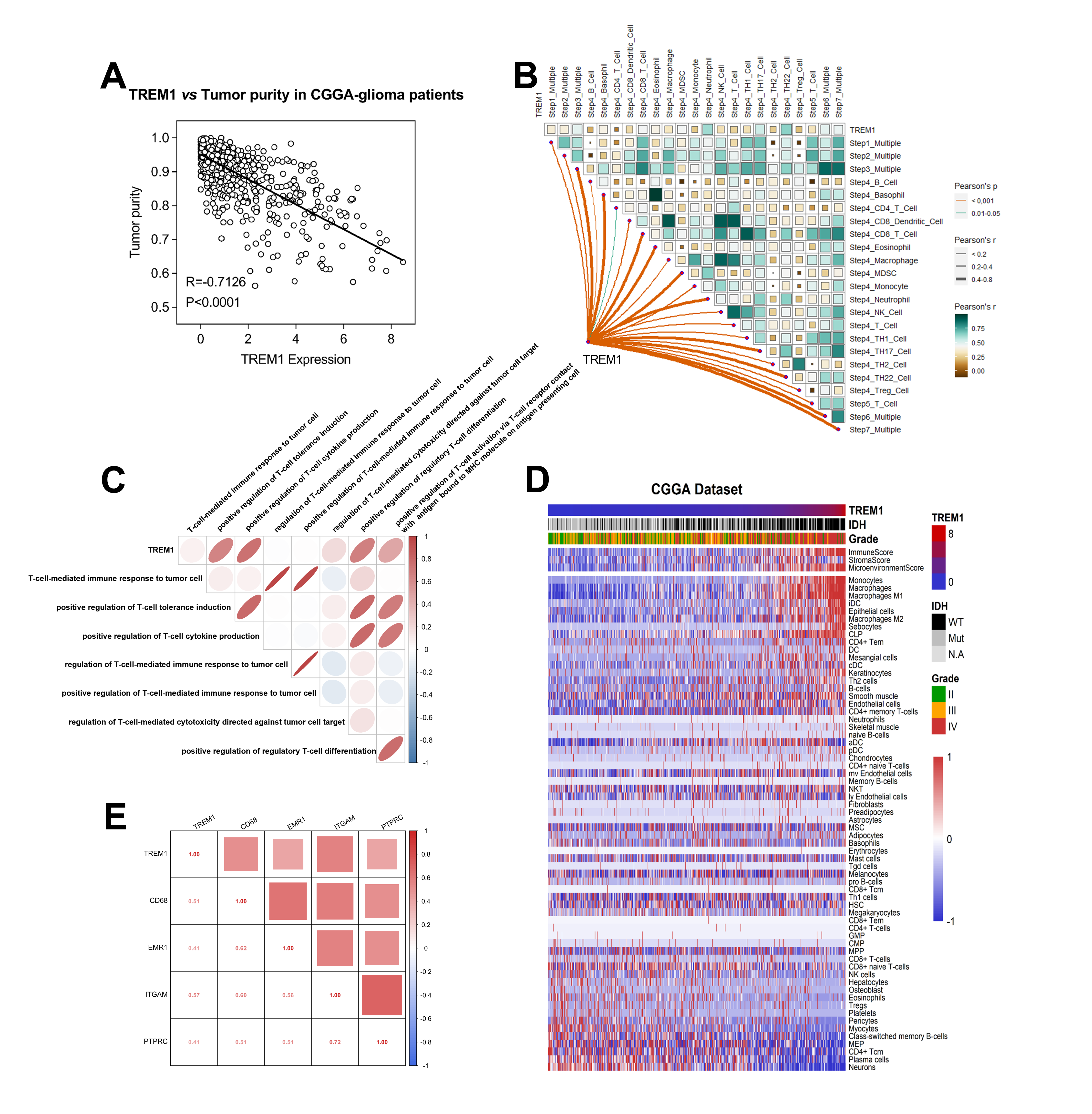

Supplement: Supplementary Figure 10 — (A) Correlation analysis between TREM1 expression levels and tumor purity in the CGGA database. (B) Correlation between TREM1 and the steps of the cancer immunity cycle in the CGGA database. (C) Relationship between TREM expression levels and T-cell-specific immunity in the CGGA database. (D) Heatmaps showing the relationship between TREM1 expression and immune and stromal cell populations in the TCGA database. Expression values were subjected to z-score normalization, with high levels shown in red and low levels shown in blue. (E) Correlation analysis between TREM1 and macrophage marker genes in the CGGA database. [file Image_10.tif]

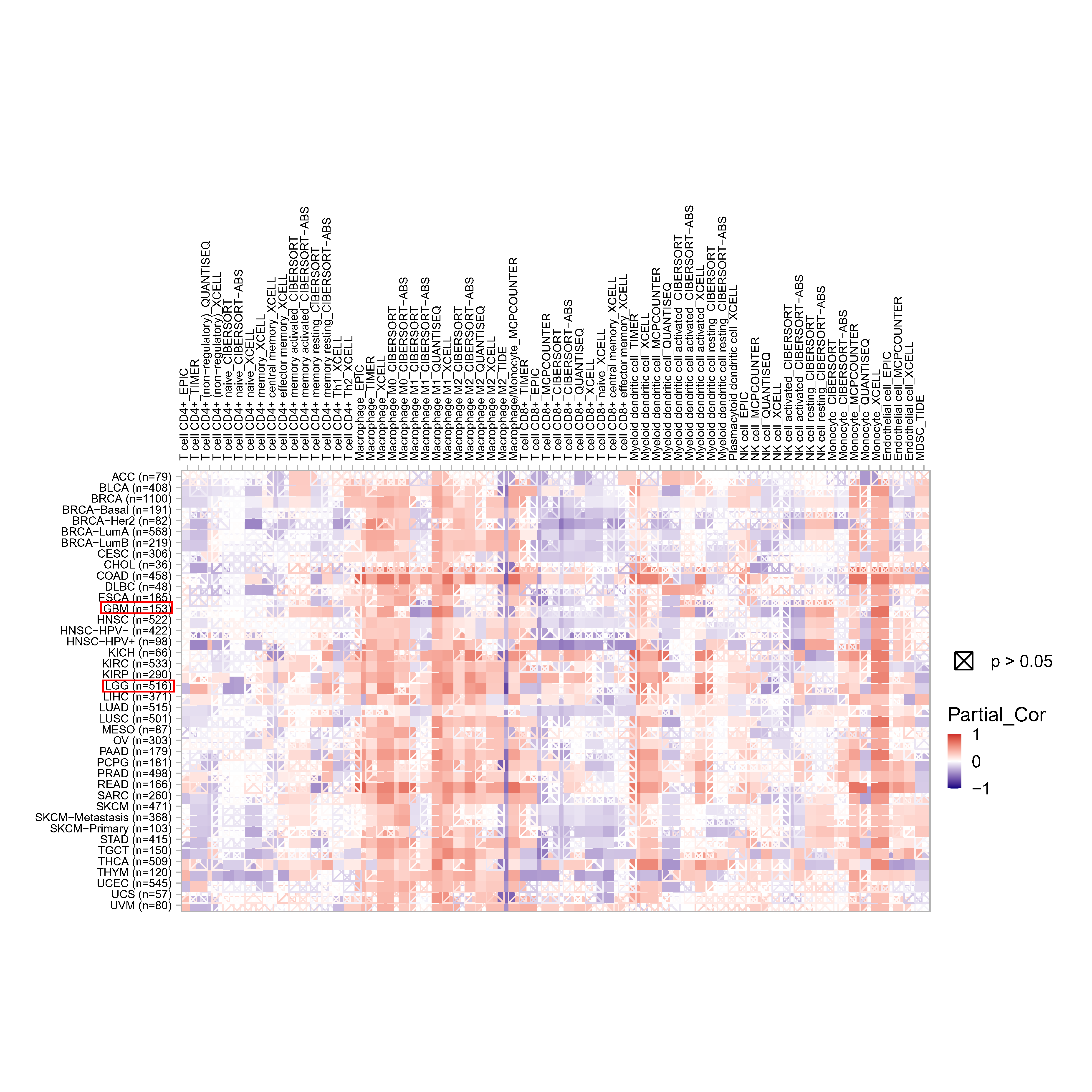

Supplement: Supplementary Figure 11 — Correlation analysis between TREM1 and nontumor cells in the tumor microenvironment across human cancers in the TCGA database. [file Image_11.tiff]
