# Supplementary material for: Molecular and immunological features of TREM1 and its emergence as a prognostic indicator in glioma
Source: Front Immunol. 2024 Feb 2;15:1324010. doi: 10.3389/fimmu.2024.1324010 (PMC10869492; doi:10.3389/fimmu.2024.1324010)
Supplement: Supplementary file 14 [file Table_1.docx]

**Table S1. Univariate and multivariate Cox regression of TREM1 expression for overall survival in CGGA glioma patients.**

| Variable | Univariate Cox Regression | | Multivariate Cox Regression | |
| --- | --- | --- | --- | --- |
|  | HR (95 % CI) | P | HR (95 % CI) | P |
| TREM1  Increasing Expression | 1.282(1.214-1.352) | 0.000 | 1.046(1.019-1.117) | 0.045 |
| Grade  GBM *vs* LGG | 4.177(3.336-5.232) | 0.000 | 2.641(1.963-3.553) | 0.000 |
| Gender  Female *vs* Male | 1.037(0.834-1.288) | 0.744 |  |  |
| Age  Increasing Years | 1.026(1.017-1.035) | 0.000 | 1.006(0.997-1.015) | 0.172 |
| IDH status  WT *vs* Mut | 3.306(2.645-4.133) | 0.000 | 1.914(1.453-2.521） | 0.000 |
| Radiotherapy  No *vs* Yes | 1.358(0.999-1.844) | 0.051 | 0.981(0.703-1.369) | 0.981 |
| Chemotherapy  No *vs* Yes | 1.541(1.175-2.022) | 0.002 | 0.950(0.701-1.288) | 0.743 |

HR, hazards ratio; CI, confidence interval.

**Table S2. Univariate and multivariate Cox regression of TREM1 expression for overall survival in TCGA glioma patients.**

| Variable | Univariate Cox Regression | | Multivariate Cox Regression | |
| --- | --- | --- | --- | --- |
|  | HR (95 % CI) | P | HR (95 % CI) | P |
| TREM1  Increasing Expression | 1.793(1.622-1.981) | 0.000 | 1.121(0.992-1.266) | 0.066 |
| Grade  GBM *vs* LGG | 9.423(7.024-12.643) | 0.000 | 1.850(1.254-2.729) | 0.002 |
| Gender  Female *vs* Male | 1.166(0.885-1.537) | 0.275 |  |  |
| Age  Increasing Years | 1.070(1.059-1.081) | 0.000 | 1.035(1.022-1.048) | 0.000 |
| IDH status  WT *vs* Mut | 10.881(7.966-14.861) | 0.000 | 3.500(2.205-5.554) | 0.000 |
| 1p/19q status  Non-codel *vs* Codel | 0.215(0.134-0.346) | 0.000 | 0.607(0.343-1.073) | 0.086 |

HR, hazards ratio; CI, confidence interval.
